# Design and Development of a Battery State of Health Estimation Model for Efficient Battery Monitoring Systems

**DOI:** 10.3390/s22124444

**Published:** 2022-06-12

**Authors:** Hyoung Sun Choi, Jin Woo Choi, Taeg Keun Whangbo

**Affiliations:** 1 Department of IT Convergence Engineering, Gachon University, Sujeong-gu, Seongnam-si 461-701, Gyeonggi-do, Korea; hschoi@gachon.ac.kr; 2 Cultural Contents Technology Institute, Gachon University, Sujeong-gu, Seongnam-si 461-701, Gyeonggi-do, Korea; jwchoi@gachon.ac.kr; 3 Department of Computer Engineering, Gachon University, Sujeong-gu, Seongnam-si 461-701, Gyeonggi-do, Korea

**Keywords:** uninterruptible power supply, battery management system, SoH, clustering, recurrent neural network, LSTM

## Abstract

An uninterruptible power supply (UPS) is a device that can continuously supply power for a certain period when a power outage occurs. UPS devices are used by national institutions, hospitals, and servers, and are located in numerous public places that require continuous power. However, maintaining such devices in good condition requires periodic maintenance at specific time points. Efficient monitoring can currently be achieved using a battery management system (BMS). However, most BMSs are administrator-centered. If the administrator is not careful, it becomes difficult to accurately grasp the data trend of each battery cell, which in turn can lead to a leakage or heat explosion of the cell. In this study, a deep-learning-based intelligent model that can predict battery life, known as the state of health (SoH), is investigated for the efficient operation of a BMS applied to a lithium-based UPS device.

## 1. Introduction

An uninterruptible power supply (UPS) is a device providing emergency power during an outage [1]. A battery is a central component of a UPS that stores and supplies power when an outage occurs. Most UPS devices are composed of inexpensive lead–acid batteries. However, lithium-ion batteries have recently become competitive for use in various products, and their application has become widespread in recent years [2]. The advantages of lithium-ion batteries are as follows. First, their lifespan is more than twice that of a lead–acid battery. Second, having twice the energy density, they require a small area and are thus easy to install. Third, they charge quickly and produce a high output. Finally, when managing a UPS device, a battery management system (BMS) is used to enable online monitoring and control, regardless of the space and time required [3,4]. A BMS is used to identify the quality of each cell and diagnose the aging condition by measuring and analyzing the characteristics of the battery’s internal resistance, cell voltage, and surface temperature of the battery’s cathode terminal.

Previously, a method of measuring cell voltage was mainly used for spare power batteries, but sound conditions such as age deterioration and performance degradation of each cell could not be accurately identified. In case of sealed batteries, the remaining capacity or sound state of each battery cell can only be accurately measured through the actual load discharge test, but it requires a lot of manpower and test costs, with risk factors such as short circuit causing unexpected accidents.

Against this backdrop, BMSs have been developed and the state of batteries can be monitored in real time by analytical software.

If a BMS is not employed, the UPS device can experience different problems. Notably, owing to the early aging of certain batteries, there can be a reduction in the duration of continuous power supply. Moreover, accidents can occur from a leakage or heat explosion of a cell. Another problem is a waste of resources after replacing the entire battery. A BMS was therefore introduced into a UPS system to compensate for these problems.

Furthermore, some studies have focused on the accurate measurement and monitoring of battery cells using a BMS [5,6]. However, some research gaps in the state of the art remain unaddressed. For instance, most existing battery cells require an accurate diagnosis by a manager. In addition, the warning alarm threshold is set as a percentage of the absolute or initial value, which can result in a false alarm if noisy data are present. Managing the individual data trends of battery cells is a challenge. Because they are driven by an absolute value, maintaining lithium-ion batteries is difficult, owing to a significant deviation in the resistance value. To solve these problems using artificial intelligence, different deep learning models can potentially achieve an efficient BMS for application in a lithium-ion-based UPS device.

In this paper, an intelligent monitoring system is introduced that tracks each individual battery cell for the efficient management and prediction of false alarms. Cell-level management allows the replacement of individual cells with an state of health(SoH) below the designated threshold. This study used a real dataset of UPS devices installed in a partnership company. The dataset, coupled with a recurrent neural network (RNN) and long short-term memory (LSTM), helps predict false alarms and supports the efficient monitoring of the BMS.

The remainder of this paper is organized as follows: Section 2 provides an overview of current state-of-the-art BMS approaches and highlights existing gaps in this field of research. Section 3 describes the proposed methodology and the different processes applied to the dataset. Section 4 describes the implementation model and provides an overview of the technology stack used to develop the proposed system. Section 5 discusses the results. Finally, Section 6 provides some concluding remarks and identifies future directions of research.

## 2. Related Work

Various efforts have recently been made to predict the SoH of a battery. In particular, because of their high energy level, high power density, and longevity, lithium-ion batteries are used as the main power sources in electric vehicles, mobile phones, and UPS devices [7]. However, lithium-ion batteries degrade owing to their nature, resulting in changes in their electrochemical characteristics, that is, a decrease in the discharge capacity and an increase in the internal resistance [8]. A BMS is essential for efficient monitoring, and studies are being conducted that combine artificial neural network techniques with predictions of temperature and nonlinear data.

Chaoui et al. [9] presented the application of a dynamically driven recurrent network in an online electric vehicle battery analysis. The proposed technique only requires certain features to accurately estimate the SoH of a battery, such as the battery voltage, charge/discharge currents, and ambient temperature variations. The main purpose of this research was to develop a suitable and effective method for applying a BMS without knowing the internal parameters of the battery.

Kwon et al. [10] presented an algorithm for SoH prediction using LSTM for rechargeable lithium-ion batteries used in electric vehicles. The main purpose of this study was to achieve a better performance in comparison to an RNN-based SoH prediction.

Li et al. [11] presented a multistep-ahead thermal warning network based on temperature detection. They proposed the use of real-time measurements to predict whether the core temperature of a lithium-ion battery energy storage system will reach a critical value within the following time window.

## 3. Proposed Methodology

The dataset used in this study is a collection of data extracted from a BMS, following the IEEE Std 1491-2012 rules, used to monitor a lithium-ion-based UPS device (4-volt) [12]. For this research, five features were used: resistance, voltage, temperature, key time, and number of cells. The data on these attributes were stored in the form of CSV files. One CSV file is referred to as 1 BANK, which consists of 96 battery cells and measures data every 12 h. The experiment was conducted by observing 240 BANK datasets.

### 3.1. Preprocessing Method

**Figure 1:**
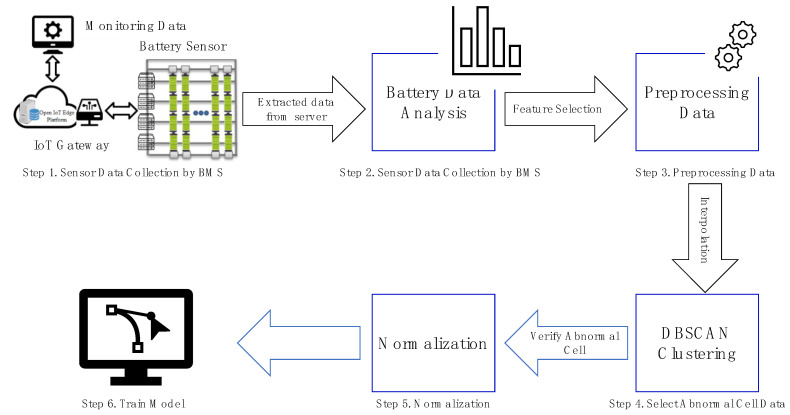
Method flow diagram.

**Figure 2:**
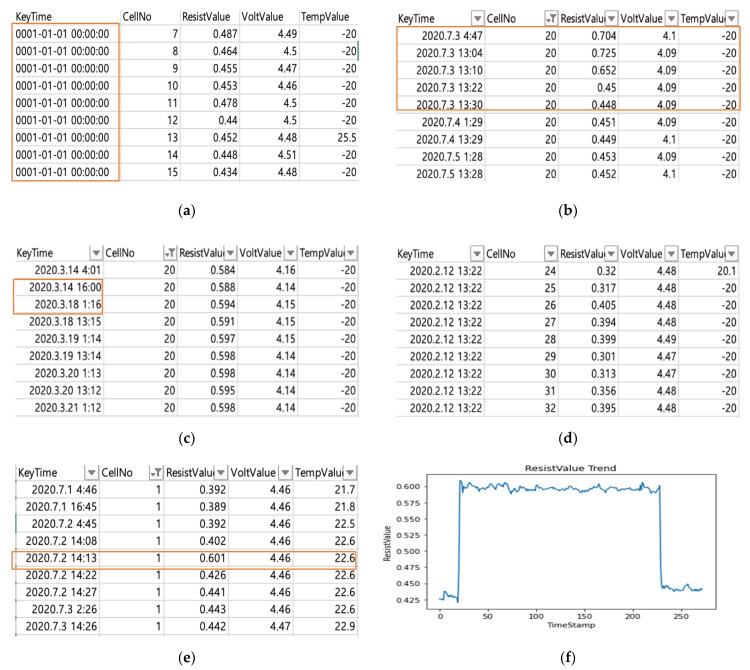
Steps for data preprocessing. (**a**) Step 1. Delete KeyTime(DateTime) error; (**b**) Step 2. Delete measurements more than twice a day; (**c**) Step 3. Fill in missing data; (**d**) Step 4. Fill in missing TempValue; (**e**) Step 5. Delete outlier data; (**f**) Step 6. Divide different trending cells.

The overall flow of the preprocessing process is shown in Figure 1. The BMS collects data from the sensors. After extracting a certain period of data from the BMS, five features were analyzed: ResistValue, VoltValue, TempValue, KeyTime, and CellNo. Figure 2 lists the order of data preprocessing. First, to obtain continuous data, the KeyTime error and data measured more than twice a day were deleted. At this time, if the data were measured more than twice a day, it is owing to an intervention by the administrator, leaving only the last stabled data and one datum which has an interval of 12 h from the stabled data. Missing data were generated through an interpolation. Similarly, owing to sensor errors, data were generated by the interpolation of unmeasured values. Subsequently, to eliminate the training of false alarm data, the outlier data were deleted, and the cells were separated if the average internal resistance changed owing to the intervention of the administrator.

After completing data preprocessing, an abnormal cell was selected for training through Density-Based Spatial Clustering of Applications with Noise(DBSCAN) clustering. To improve the performance of the model, normalization was conducted using MinMaxScaler. The method for verifying abnormal cells is described in Section 3.3.

**Figure 3:**
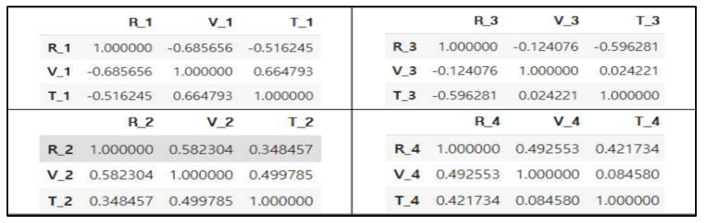
Correlation for each cell.

Before starting the experiment, we used the correlation of the given dataset for the feature selection, as shown in Figure 3 [13]. Based on this, although different features were used for predicting the resistance, the use of only one feature showed the highest performance.

**Figure 4:**
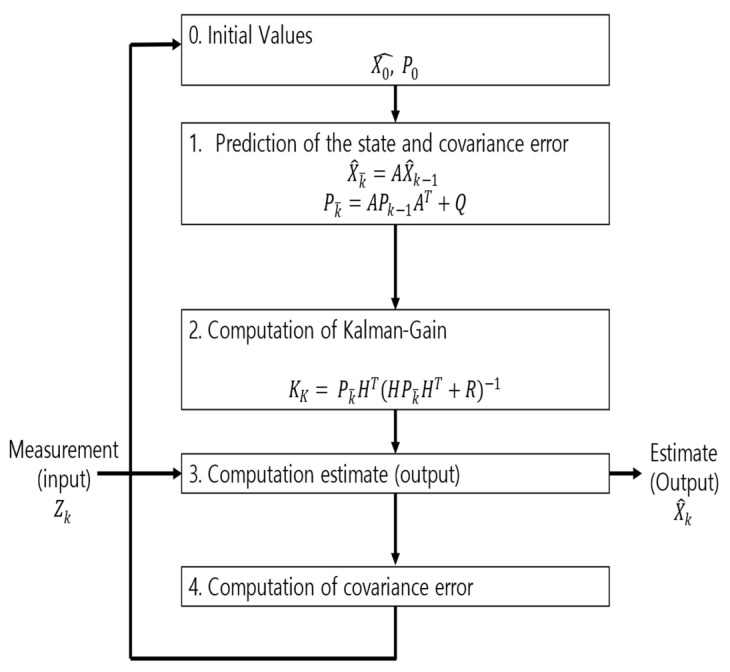
Kalman filter algorithm.

Time-series data generate noise in certain parts, and there is a limit to improving the performance of the model when training data include such noise [14]. Consequently, a Kalman filter, which is widely applied in signal processing and time-series analysis, is used [15]. A Kalman filter is a recursion filter that estimates the state based on noise-containing data and estimates the distribution of the current state variable based on previous measurements. In addition, because the computation process is fast, it is suitable for real-time problems. Moreover, because there is no need to maintain records other than the previous state, it is ideal for systems that require continuous updates. Figure 4 and Table 1 show the applied Kalman filter algorithm and its description.

Therefore, the amount of noise was minimized by utilizing a Kalman filter when considering the mix of noise data at the measurement time. As shown in Table 2, we used different hyperparameter values to appropriately smooth the data without deviating significantly from the original data.

### 3.2. Model Selection

In selecting the abnormal cells used for training, it is time-consuming to apply all cells to the model. Typically, cells that have increased resistance value cause the battery to have higher temperature and cause a drop of the voltage. Then, eventually, the battery is cut off, leaving energy behind [16]. Consequently, we set the threshold for the abnormal cell as increase of 150% of the average of monthly resistance value.

(1) $$Threshold=\frac{\sum \mathrm{Resistance}}{\mathrm{len}(\mathrm{Monthly}\;\mathrm{Data})}\times 150\%\;$$

Therefore, for the next step, specific cells that had reached the end of their SoH were extracted using clustering. There are many clustering approaches, including a support vector machine [17], K-means clustering [18], and Density-Based Spatial Clustering of Applications with Noise(DBSCAN) clustering [19]. DBSCAN is typically used for the density-based spatial clustering of noisy datasets. The main idea here is that a minimum number of points (minPts) should be included in the given radial (epsilon) neighborhood of each clustering object, which indicates that the density of the neighborhood must exceed a certain threshold. For the parameters of DBSCAN are described in Table 3.

**Figure 5:**
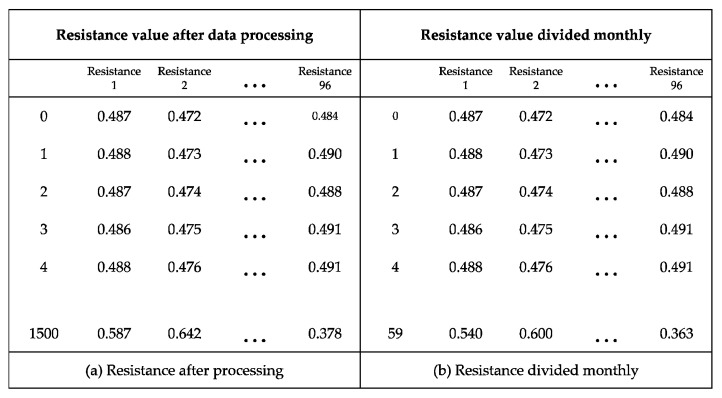
Results of processing clustering.

As mentioned earlier, the dataset consists of resistance, temperature, and voltage, as shown in Table 4. Only resistance was used for clustering, and thus the preprocessing data remain, as shown in Figure 5a. Finally, the dataset was divided into monthly units, as shown in Figure 5b.

After dividing the dataset by month and comparing the clustering results of the next month with those of the first month, it is possible that an abnormal cell exists if the results differ.

### 3.3. Verification of DBSCAN Clustering

As mentioned earlier, when resistance value increases by more than 150% compared with the initial value, the cell is considered abnormal. Therefore, we first set a value that is 150% more than the mean of the resistance as the threshold.

**Figure 6:**
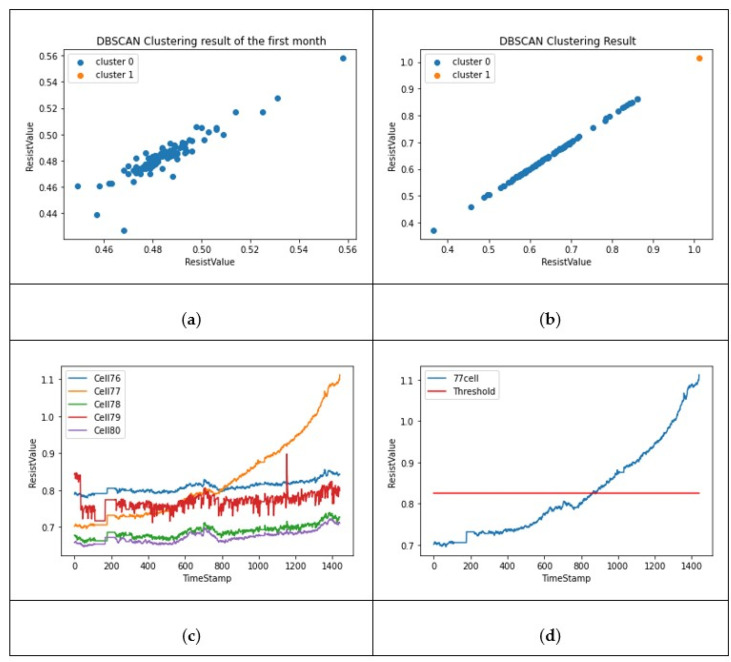
(**a**) Each cell positioned before clustering; (**b**) clustered cells result; (**c**) indicates the trend of resist value from cells 76~80; (**d**) indicates resist value trend for cell 77.

When a cell is clustered as a dangerous cell, it is possible to verify whether it can be correctly distinguished by comparing the resistance with the threshold values. As a result of the performance, cell no. 77 was classified into another cluster, as shown in Figure 6a,b. By printing the data trends, as shown in Figure 6c,d, it can be clearly verified whether the cell is abnormal.

### 3.4. Dataset and Normalization

**Figure 7:**
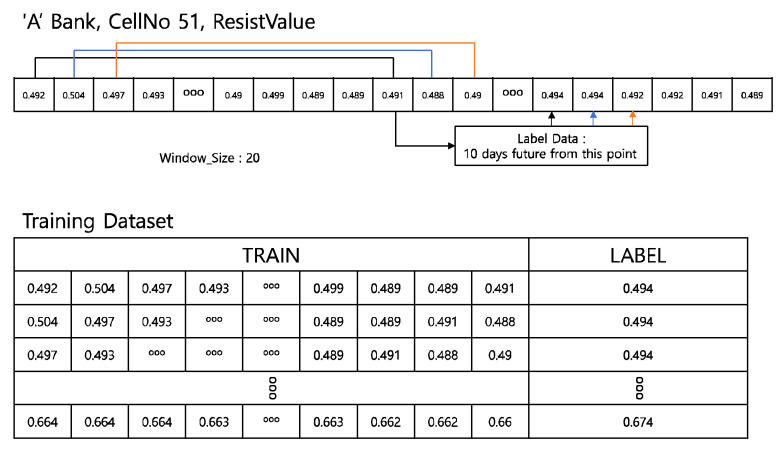
Training dataset.

Although deep-learning-based models achieve a high accuracy and performance, the amount and quality of the training data are important. Therefore, only cells classified as dangerous are extracted. The training dataset is created as shown in Figure 7.

Each extracted cell is configured with a different resistance and measured time, and applying different weights during the model training can decrease performance. Therefore, using MinMaxScaler [20], a type of normalization, to proceed with the training, the data were converted into values between 0 and 1. In Equation (2), *X* stands for each value of the data.

(2) $$MinMaxScaler=\frac{X-\min(X)}{\max(X)-\min(X)}$$

## 4. Implementation Model

Models optimized for time-series data were used in this study. Typically, RNN and LSTM are used for time-series prediction [21]. For forecasting, data have to be divided by windows, such as in Figure 7, and then it is decided how many data will be trained and which timestamp will be labeled. If the window size is large, more information will be trained, but it can cause a decrease of the sensitivity of the model.

**Figure 8:**
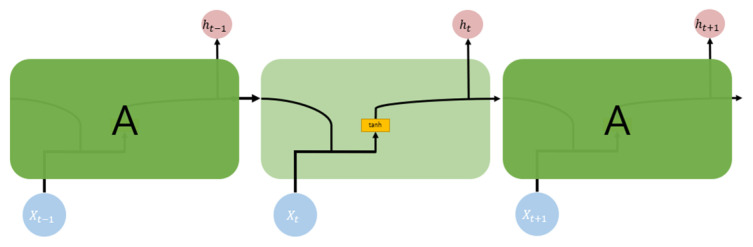
RNN block.

An RNN [22] is a type of artificial neural network in which hidden layers are connected to the edge to form a circulatory structure. Regardless of the sequence length, this has the advantage of creating a structure with flexible inputs and outputs. However, if the distance between the relevant information and the point where the information is used in the RNN is large, the gradient gradually disappears as it reverses. This is called the problem of long-term dependency, which results in a decrease in the training performance. Figure 8 shows the detailed structure of an RNN.

**Figure 9:**
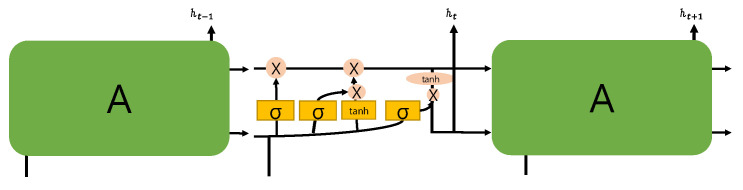
LSTM block.

An LSTM [23] is a network designed to solve the long-term dependency problem of an RNN, which is mainly used in natural language processing (NLP). In an RNN-based cell, as shown in Figure 8, the current input and output sequence of the hidden state is used as input, whereas in the case of an LSTM, as shown in Figure 9, the cell has a more complex structure. The forget gate f_t adjusts those data that will be left from the previous state. Because the output range of a sigmoid function is between 0 and 1, it is possible to forget the information of the last state if the value is 0 and to fully remember the information of the previous state if the value is 1. In addition, there is a gate that updates the state based on the current input and an output gate for controlling information that will go beyond the next step; therefore, previous information may remain for a long time.

**Figure 10:**
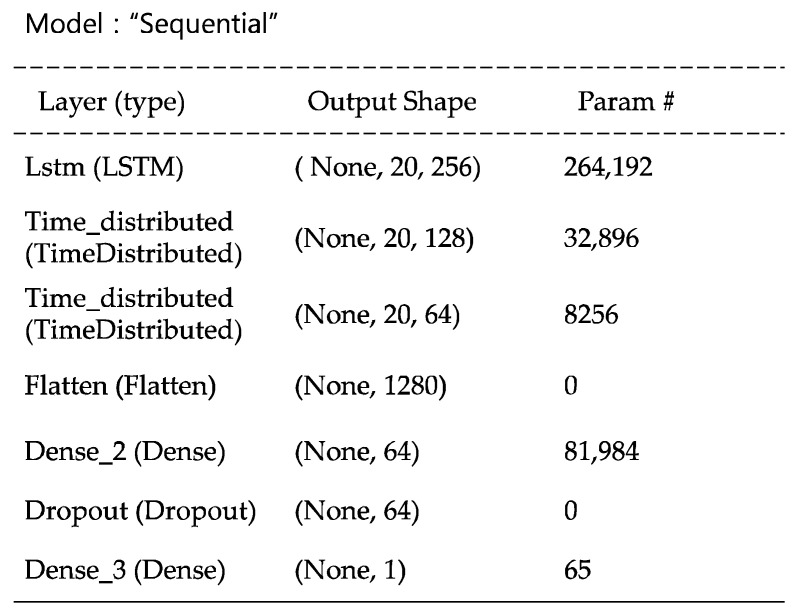
RvNN model architecture.

A recursive neural network (RvNN) is a generalized version of a recurrent network. It applies the same set of weights recursively over a structured input to produce a structured prediction over variable-sized input structures, or a scalar prediction by traversing a given structure in topological order. RNNs have been successfully used in learning sequences and tree structures in NLP, mainly phrase and sentence continuous representations, based on word embedding [24]. The suggested model for this research is described by Figure 10. By using Keras early stopping, 21 epochs and 26 min were used for training.

## 5. Results

Artificial neural network models are influenced by the number and quality of the datasets. As mentioned in various studies, based on the results of a data correlation, the internal resistance is mostly related to temperature. However, in this study, the temperature was generated through an interpolation, which reduces the performance of the model. Therefore, we applied only the internal resistance to predict the future value for the SoH of a single cell. Table 5 shows the programming environment. A total of 23,040 (240 BANK × 96 cells) cells and 28 abnormal cells were used for training. Five abnormal cells were used for testing.

To compare the accuracy of the model, the RMSE and R2 score were used. In Equations (3) and (4), N is the length of the data, $y_{i}$ is the actual value, ${\bar{y}}_{i}$ is the average of the actual value, ${\hat{y}}_{i}$ is the predicted value, SSE is the explained sum of squares, SSR is the residual sum of squares, and SST is the total sum of squares. Based on the results in Table 6, the RvNN achieved the highest performance.

(3) $$RMSE=\sqrt{\frac{1}{N}\sum_{i=1}^{N}{\left(y_{i}-{\hat{y}}_{i}\right)}^{2}}$$

(4) $$R^{2}=\frac{SSE}{SST}=1-\frac{SSR}{SST}=1-\frac{\sum_{i=1}^{n}{\left(y_{i}-{\hat{y}}_{i}\right)}^{2}}{\sum_{i=1}^{n}{\left(y_{i}-{\bar{y}}_{i}\right)}^{2}}=1-\frac{MSE}{Var(y)}$$

## 6. Conclusions

In this paper, we designed and developed a battery’s state of health estimation for a BMS. It aims to use artificial intelligence to detect false alarm from the BMS and to predict and identify each battery cell’s state of health by using time-series data consisting of resistance value and time. For designing the model, an RvNN network was selected among deep learning models. 

A BMS is used to efficiently monitor UPS in real time, but most of the BMSs have difficulty with precise measurement of sealed batteries. In response, a partnership company proposed a BMS with a clamp sensor to overcome shortcomings and enable to monitor each cell in real time, but a BMS is a very manager-dependent technique. In addition, sensor errors or ambient noise can cause an outlier, resulting in a false alarm. This is why AI was introduced for this study.

For training, time-series data were used in this study. For time-series data, it is well known that RNN and LSTM have the highest performance. From a novelty standpoint, we applied an RvNN to time-series data, which is typically used for sequence labeling problems, and it can minimize the waste of resources by replacing only the abnormal cell instead of replacing the entire battery.

For the experiment, abnormal cells were extracted from the dataset using DBSCAN clustering. Since then, 80% of the dataset were used for training and the remaining 20% were used for testing. To minimize overfitting of the proposed model, we used early stop-ping and adjusted the learning rate to 0.001.

For validation, we collected abnormal cells from another server UPS which were not used in training. To verify the performance of the proposed model, different evaluation methods were used, i.e., MAE, RMSE, and R2 score. As shown in the results in Table 5, the overall average R2 score of abnormal cells RvNN 0.91, LSTM 0.83, and RNN 0.77 found that RvNN performed better than RNN and LSTM. However, RvNN took about 30% longer in training time compared with the other two models due to many repetitive operations.

In future research directions, the proposed RvNN method may be useful in predicting SoH in batteries, so it can be tried in other types of industrial environments. However, there are some points that need to be improved because of the time cost during the operation. Furthermore, if there are data that are completely different from the training data, it will show very low performance. To overcome this, studies will be conducted in consideration of proposing a model with reinforcement learning based on recursive models.

## Figures and Tables

**Table 1:** Variables for Kalman Filter.

Variable	Description
$\hat{X_{0}},\;P_{0}$	Initial value
$Z_{k}$	Measurement (input)
${\hat{X}}_{k}$	Predicted of the state
$P_{k}$	Covariance error
${\hat{X}}_{\bar{k}}$	${\hat{X}}_{k}$ predicted value
$P_{\bar{k}}$	$P_{k}$ predicted value
A	State space equation matrix
H	Observation matrix
Q	System noise
R	Measurement noise

**Table 2:** Hyperparameters used for Kalman filter.

Q	R	Plot	Explanation
0.00001	0.01	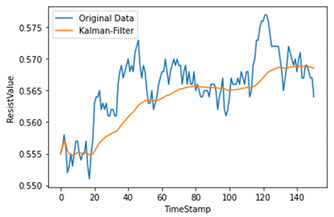	Significant differences among original data
0.00001	0.1	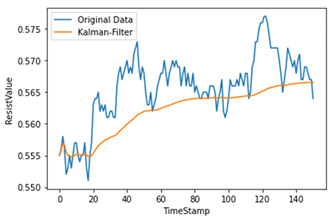	
0.0001	0.01	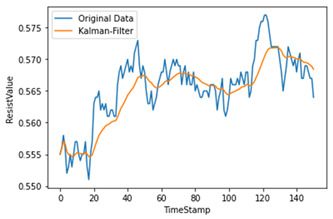	Larger Q makes the filtered data close to the original data
0.0001	0.001	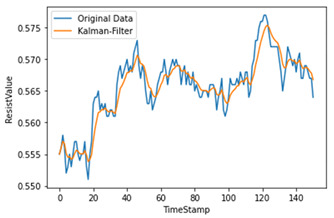	Reduces most noise data and is close to original data

**Table 3:** Overview of DBSCAN parameters.

Parameter	Explanation	Parameter Value
Epsilon	The maximum distance between two samples for one to be considered within the neighborhood of the other.	1
minPts (min_samples)	The number of samples (or total weight) in a neighborhood for a point to be considered as a core point.	5

**Table 4:** BANK data prior to clustering.

	Resistance 1	Volt 1	Temp 1	Resistance 2	Volt 2	Temp 2	…	Resistance 96	Volt 96	Temp 96
0	0.487	4.47	20.5	0.472	4.45	NaN	…	0.484	4.45	NaN
1	0.488	4.47	20.2	0.473	4.44	NaN	…	0.490	4.44	NaN
2	0.487	4.47	20.3	0.475	4.45	NaN	…	0.488	4.45	NaN
3	0.486	4.48	19.5	0.475	4.45	NaN	…	0.491	4.45	NaN
…										
1500	0.587	4.45	19.3	0.642	4.42	NaN	…	0.378	4.46	NaN

**Table 5:** Programming environment.

Library	Version
Python	3.7.12
Pandas	1.3.5
Numpy	1.21.5
Keras	2.8.0
Tensorflow	2.8.0
Scikit-Learn	1.0.2

**Table 6:** Result of each model prediction for abnormal cells.

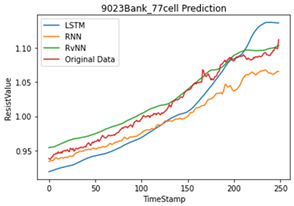	**Learning Model**	**Max Difference**	**MAE**	**RMSE**	**R^2^ Score**
	LSTM	4.424	0.023	0.025	0.871
	RvNN	1.934	0.009	0.01	0.96
	RNN	4.558	0.022	0.025	0.656
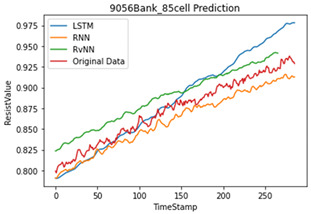	**Learning Model**	**Max Difference**	**MAE**	**RMSE**	**R^2^ Score**
	LSTM	5.113	0.017	0.022	0.843
	RvNN	2.258	0.01	0.01	0.936
	RNN	2.992	0.012	0.013	0.86
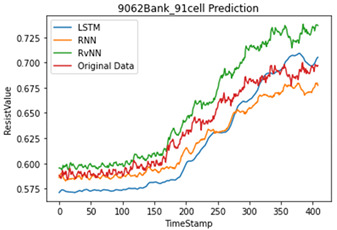	**Learning Model**	**Max Difference**	**MAE**	**RMSE**	**R^2^ Score**
	LSTM	4.760	3.271	0.035	0.789
	RvNN	2.818	0.019	0.022	0.824
	RNN	5.373	0.014	0.016	0.783

## Data Availability

Not applicable.
